# How can cry acoustics associate newborns’ distress levels with neurophysiological and behavioral signals?

**DOI:** 10.3389/fnins.2023.1266873

**Published:** 2023-09-20

**Authors:** Ana Laguna, Sandra Pusil, Irene Acero-Pousa, Jonathan Adrián Zegarra-Valdivia, Anna Lucia Paltrinieri, Àngel Bazán, Paolo Piras, Clàudia Palomares i Perera, Oscar Garcia-Algar, Silvia Orlandi

**Affiliations:** ^1^Zoundream AG, Basel, Switzerland; ^2^Facultad de Medicina, Universidad Señor de Sipán, Chiclayo, Peru; ^3^Global Brain Health Institute, University of California, San Francisco, San Francisco, CA, United States; ^4^Achucarro Basque Center for Neuroscience, Leioa, Spain; ^5^Neonatology Department, Barcelona Center for Maternal-Fetal and Neonatal Medicine (BCNatal), Hospital Clínic, Universitat de Barcelona, Barcelona, Spain; ^6^Department de Cirurgia I Especialitats Mèdico-Quirúrgiques, Universitat de Barcelona, Barcelona, Spain; ^7^Department of Electrical, Electronic and Information Engineering “Guglielmo Marconi” (DEI), University of Bologna, Bologna, Italy; ^8^Health Sciences and Technologies Interdepartmental Center for Industrial Research (CIRI-SDV), University of Bologna, Bologna, Italy

**Keywords:** cry acoustic, EEG, NIRS, newborns, distress, body language

## Abstract

**Introduction:**

Even though infant crying is a common phenomenon in humans’ early life, it is still a challenge for researchers to properly understand it as a reflection of complex neurophysiological functions. Our study aims to determine the association between neonatal cry acoustics with neurophysiological signals and behavioral features according to different cry distress levels of newborns.

**Methods:**

Multimodal data from 25 healthy term newborns were collected simultaneously recording infant cry vocalizations, electroencephalography (EEG), near-infrared spectroscopy (NIRS) and videos of facial expressions and body movements. Statistical analysis was conducted on this dataset to identify correlations among variables during three different infant conditions (i.e., resting, cry, and distress). A Deep Learning (DL) algorithm was used to objectively and automatically evaluate the level of cry distress in infants.

**Results:**

We found correlations between most of the features extracted from the signals depending on the infant’s arousal state, among them: fundamental frequency (F0), brain activity (delta, theta, and alpha frequency bands), cerebral and body oxygenation, heart rate, facial tension, and body rigidity. Additionally, these associations reinforce that what is occurring at an acoustic level can be characterized by behavioral and neurophysiological patterns. Finally, the DL audio model developed was able to classify the different levels of distress achieving 93% accuracy.

**Conclusion:**

Our findings strengthen the potential of crying as a biomarker evidencing the physical, emotional and health status of the infant becoming a crucial tool for caregivers and clinicians.

## 1. Introduction

Human infants’ communication through crying shares its evolutionary basis with animal distress calls and is based on their physical and emotional state (Friedlander, 2006) under the solicitation of help-provisioning and nurturing behavior (Bylsma et al., 2019). Thus, newborn crying may function as a distant early warning signal or “biological siren” (Golub and Corwin, 1985) that engages the caregiver’s attention and demands their return to the infant’s side (Porges et al., 1994). In contrast with discrete signals, which manifest little variation in duration or intensity, infant crying fits much better in the concept of graded signals that convey degrees of distress and that reflect the intensity and duration of the eliciting stimulus. Hence, the sounds of crying convey a level of distress and/or urgency of need (Friedlander, 2006).

Research studies published in the last few years focused on the identification of the acoustic cry features (LaGasse et al., 2005; Manfredi et al., 2018) to study the well-being of the newborns, neonatal diseases (Lawford et al., 2022) and neurodevelopmental disorders (Esposito and Venuti, 2010) through signal processing and Artificial Intelligence (AI) techniques (Farsaie Alaie and Tadj, 2012; Zabidi et al., 2018; Morelli et al., 2021). Acoustic cry features include fundamental frequency (F0) (Porter et al., 1988), resonance frequencies (F1-F3) related to vocal tract maturation, parameters of vibrato rate and extent (jitter and shimmer), and noise levels (Wermke et al., 2002). While infant cry analysis has been extensively studied, limited research has explored the acoustic characteristics of distinct cry states. Existing studies primarily focus on pain cries, which exhibit greater variations in F0 (Bellieni et al., 2004; Zamzmi et al., 2018). Additionally, several recent studies focused on the development of AI tools in neonatal medicine highlighting its potential as a powerful tool to support clinical decision making, personalized care, precise prognostics, and enhance patient safety (Kwok et al., 2022).

The production of infant cry vocalizations is a complex process requiring coordinated brain activity and involvement of the central nervous system, which includes laryngeal activity, respiratory movements, and supralaryngeal (articulatory) activity under parasympathetic vagal control (Bylsma et al., 2019). In infant crying literature, the vagus nerve plays a crucial role in influencing acoustics, particularly the fundamental frequency (F0) (Porter et al., 1988; Porges et al., 1994). F0 increases are primarily influenced by vocal fold tension, which is modulated by the contraction of laryngeal muscles innervated by sympathetic and parasympathetic (vagal) inputs from the autonomic nervous system. Specifically, vagal input from the right nucleus ambiguus of the medulla inhibits vocal muscle contraction, leading to lower vagal activity resulting in higher F0 during infant crying (Vogt and Barbas, 1988). This vagal control of the larynx not only affects vocal intonation but also influences heart rate and reflects specific emotional states. Distress and urgency in infant cries are acoustically evident, alongside facial expressions, vagal tone, cortisol levels, bodily movements, and brain activity (Porges et al., 1994).

Several studies have explored the relationship between vagal function, F0 in infant crying, and the polyvagal hypothesis in typically developing infants (Porter et al., 1988; Shinya et al., 2016). Porter et al. (1988) reported the correlation between cardiac vagal tone and the F0 of crying in term newborns who experienced a circumcision procedure. In this case, the vagal tone, measured by respiratory sinus arrhythmia (RSA), was significantly reduced during the severely stressful procedure, and the reduction was paralleled by a significant increase in the F0 of the pain infants’ cries.

Regarding brain activity during crying, few studies (Vogt and Barbas, 1988) suggest the brain stem model of crying, supported by animal studies and human cases that focus on the implication of basal ganglia, cerebellum, and brainstem in anencephalic infants (Newman, 2007). Furthermore, primate studies have suggested the implication of bilateral cingulate cortex, limbic system-anterior part, and hippocampal gyri in crying vocalization (Kaada, 1951). Nonetheless, the localization of brain regions associated with vocalization and crying in human infants remains a difficult task (Vogt and Barbas, 1988).

Nowadays, brain signals can be non-invasively and continuously measured by near-infrared spectroscopy (NIRS) and/or electroencephalography (EEG). There are few studies (Futagi et al., 1998; Manfredi et al., 2008) related to the brain activity associated with the newborn’s cry acoustic features. Manfredi et al. (2008) show that the blood oxygenation level in preterm newborns is affected by stress caused by the effort required during crying. Considering EEG, Futagi et al. (1998) analyzed the neurophysiological activity evoked in the theta band of 29 infants with EEG, finding that the cry elicited a posterior theta brain activity.

In summary, scarce research has been accomplished to understand infant cry by concurrently assessing diverse newborn’s measures. Thus, this manuscript presents an exploratory study where a multimodal data collection has been conducted to understand if cry, EEG, NIRS, facial expressions and body movements have associations among them and with newborns’ distress conditions.

First, our aim was to characterize and compare the different cry distress levels of newborns using the features mentioned above. Second, to determine the associations between cry acoustics with the neurophysiological and behavioral features depending on the level of cry distress of the newborn and estimate their concordance. Finally, our third aim was to build a DL audio classification algorithm to demonstrate the objectivity of qualitative audio annotation and to automatically evaluate the level of cry distress in infants to prove its potential as a signal biomarker supporting clinicians on the assessment of the infant’s well-being. Therefore, we hypothesized that what is occurring at an acoustic level can also be characterized and associated with behavioral and brain neurophysiological patterns underlying the human infant cry.

To our knowledge, this is the first study that uses cry audio analysis as a potential clinical biomarker of newborns’ distress state, cross-validated with behavioral and brain signal analysis in newborns being a valuable tool in the future neonatology.

## 2. Methods

### 2.1. Participants

Twenty-five healthy full-term newborns mean gestational week 39.24 ± 7.82, recording age 7.27 ± 11.40 days after birth, 15 males/10 females, head circumference 34.08 ± 1.43, birth weight 3020.20 ± 324.11, Apgar Score at one 8.84 ± 0.85, five 9.79 ± 0.66 and 10 min 9.94 ± 0.24, umbilical cord PH (pHAU) 7.23 ± 0.07, type of delivery: eutocic (*n* = 18)–dystocic (*n* = 7), were recruited at the Hospital Clínic Barcelona (Spain). Infants had been assessed by board-certified neonatologists and diagnosed as healthy term newborns with no major congenital abnormalities or illness since birth. More details are provided in Supplementary material.

### 2.2. Procedure

Data collection was performed during the standard routine of newborn nursing (before and post feeding, etc.). As such one session was conducted with each neonate. Synchronized EEG, NIRS, audio, and video recordings were acquired for each newborn, who was lying down comfortably in a cot in the hospital maternity ward.

Different cry distress levels were defined as changes in the newborn’s status generated by uncomfortable scenarios (i.e., fuzziness, stress, pain, etc.), yielding in the following conditions: resting, cry and distress. Through the paper the words cry distress levels will be used to express the different cry conditions studied as mentioned before. The cry distress levels were defined also based on the outcome obtained through the COMFORT scale (Van Dijk et al., 2000; Wielenga et al., 2004).

**Figure 1:**
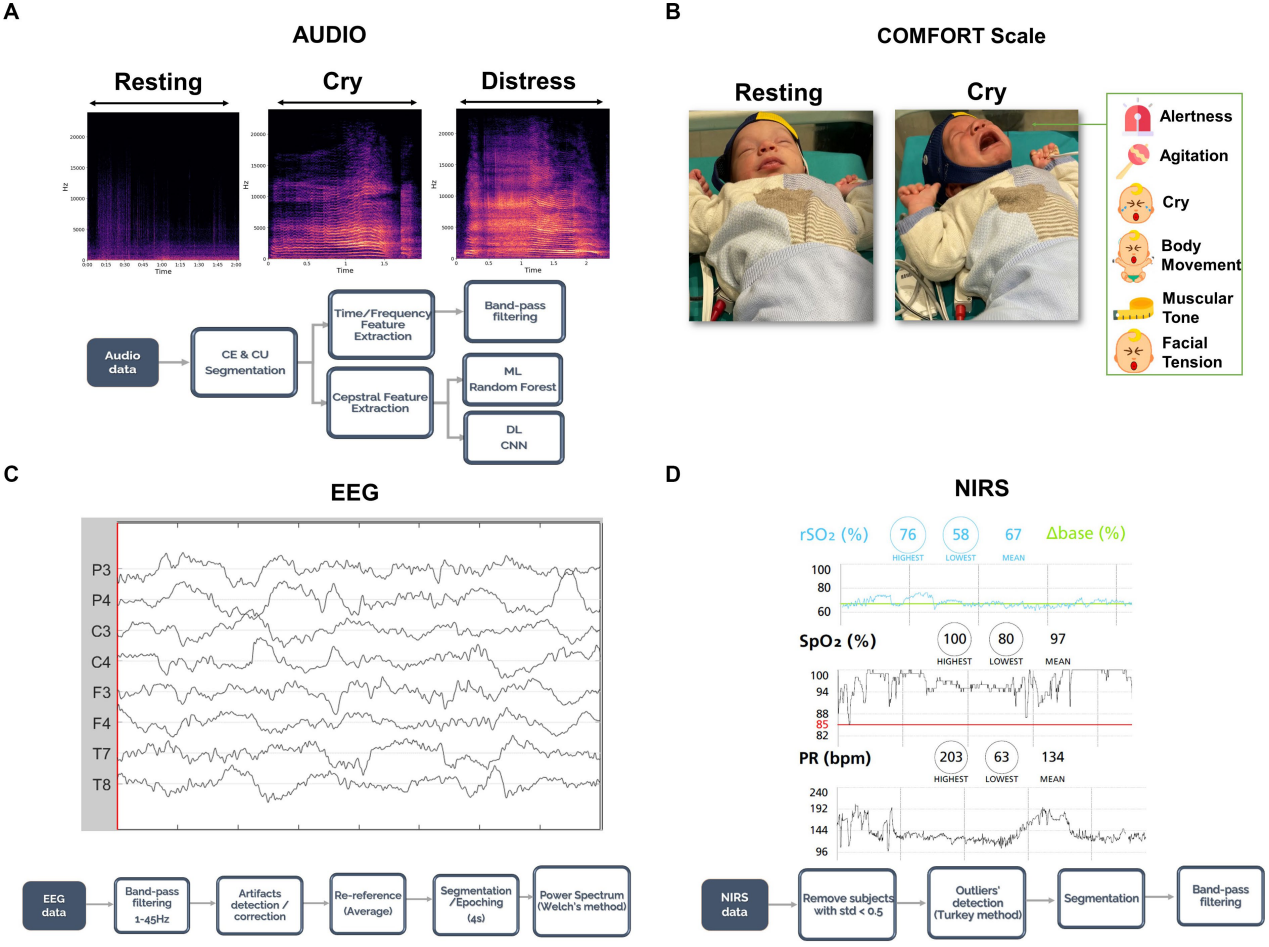
Paradigm, data acquisition, and analysis pipeline. **(A)** Audio was recorded and segmented in cry episodes and cry units depending on the distress levels of the cry. Then, time and frequency features were extracted with Praat and noise/outliers were removed with a band-pass filter. **(B)** Video was recorded for each session and the newborn’s facial expressions and body movements were assessed through the COMFORT scale. **(C)** EEG data were collected for the whole session; a preprocessing step as shown here was then applied to ensure high data quality. Lastly, clean EEG data were segmented according to the audio segmentation and the power spectrum was computed. **(D)** NIRS data were acquired for the whole session and a pre-processing pipeline as shown in this panel was followed. As for the EEG, NIRS data were segmented with the audio segmentation procedure. Consent was obtained from the family to publish the newborn’s face in the figure for publication.

To ensure proper data synchronization among diverse data sources, all devices were accurately synchronized using timestamps before each session. This synchronization was complemented by the inclusion of manual markers in every signal. The synchronization process was conducted offline using the aforementioned markers. Throughout the data collection process, two technicians per recording session were involved. They marked the occurrence of various events during data acquisition by pressing a button on each device (EEG Nëo system, NIRS-Massimo Root O3, ZOOM H1N™ manual audio recorder and video recorder) including infant crying, end of infant crying, awake states, active sleep, quiet sleep, holding the newborn, feeding, excessive movement, and more. Figure 1 shows the experimental design and overall analysis pipeline.

### 2.3. Audio analysis pipeline

#### 2.3.1. Data acquisition

Newborn crying emissions were recorded by a manual recorder (ZOOM H1N™) equipped with a unidirectional microphone, positioned at a fixed distance (30 cm) from the infant’s mouth with sampling rate Fs = 48 kHz and 24-bit resolution. Cries were never induced for the purpose of the study, as spontaneous vocalizations are part of normal infant behavior. Several audio recordings were registered during each session, to include various crying episodes, with a suitable amount of time both before and after each cry episode. During the recording, environmental noises, including human speech and noises from mediwcal machinery, were also captured. Thus, our dataset resembles that of real-world samples.

### 2.3.2. Data processing

#### 2.3.2.1. Segmentation

Audio recordings were manually segmented into cry episodes (CEs–the amount of time the infant cries in each audio recording divided by silence periods). Then, CEs were manually segmented into cry units (CUs - individual cry patterns within a CE separated by an expiration phase). Visual spectrographic analysis was carried out using iZotope RX 7 Audio Editor™. CEs and CUs were classified based on spectral content and intensity (Gustafson and Green, 1989; see Figure 1A). Two authors experts on infant cries annotation(AL, PP) individually reviewed and annotated all CEs and CUs in terms of spectrographic features and duration identifying the categories: cry and distress. Cries without unanimous agreement were excluded from further analyzes to ensure data quality throughout the whole analysis. Afterwards, the three different distress levels have been acoustically identified in every CE:

- resting: no CEs, pause or resting periods with silent audio recordings, the newborn is not crying but awake/alert state.
- cry: CEs composed by lower spectral content CUs and milder acoustical intensity.
- distress: more acoustically intense CEs that are composed of high spectral content CUs.

### 2.3.3. Feature extraction

#### 2.3.3.1. Cepstrum analysis

To prove the objectivity of qualitative annotation and the potential to automatically differentiate cry distress levels, several Machine Learning (ML) and DL algorithms were applied. The first approach used the first 13 Mel Frequency Cepstral Coefficients (MFCCs) of every CU as input features computed using the Python 3 package for audio analysis Librosa. The second approach uses spectrograms of each CU and a Convolutional Neural Network (CNN) (O’Shea and Nash, 2015) with 2D convolutional and dense layers. To prevent overfitting, pooling, and batch normalization layers were incorporated for training optimization. Both approaches utilized 80% of the samples for training the model and 20% to validate the algorithm during the learning process.

#### 2.3.3.2 Time analysis

Within CEs (cry episodes), the actual cries are not continuous vocalizations, but punctuated by inspirations and spontaneous pause or silence periods. The total duration in seconds of cry parts within the CE is defined as cryCE (amount of cry in cry episodes) while the total sum of seconds of unvoiced periods (inspirations, pauses, etc) within the CE is named as unvoicedCE (unvoiced parts in cry episodes). Percentages of cry and unvoiced parts within every CE were also computed and described as cryCE (%) and unvoicedCE (%) respectively.

#### 2.3.3.3. Frequency analysis

Audio processing of each CU was conducted through Praat software (Boersma, 2002) using a band-pass filter between 200 and 1,200 Hz to compute the F0 and a low-pass filter of 10,000 Hz to compute the spectrum (Rautava et al., 2007). Audio recordings were collected with a sampling rate of 48,000 Hz. The main frequency features include F0 and its descriptive statistics (maximum, minimum, mean, standard deviation), the resonance frequencies of the vocal tract (F1, F2, F3) along with the percentage of high pitch (F0 > = 800 Hz) (Kheddache and Tadj, 2013) and hyper-phonation (F0 > = 1000HZ) (Zeskind et al., 2011) level of the CU were computed. Other voice quality parameters related to the phonation of the vocalization are also included: local jitter (Jitter: micro-variations of the F0 measured with pitch period length deviations), local shimmer (Shimmer: amplitude deviations between pitch periods), harmonic to noise ratio (HNR, quantifies the amount of additive noise in the voice signal) (Teixeira et al., 2013).

### 2.4. Electroencephalography pipeline

#### 2.4.1. Data acquisition

Neurophysiological data were acquired using an ANT Nëo Monitor eego™ (ANT Neuro, Germany) with 8 EEG channels. The electrodes were placed according to the extended 10–20 positioning system (channels F3, F4, C3, C4, T7, T8, P3, P4) and were later re-referenced offline to the average reference. The sensor impedance was kept below 10kΩ, and EEG data were acquired at a sampling rate of 512 Hz.

#### 2.4.2. Data processing

The dataset was analyzed offline using Matlab r2022a with the Brainstorm Toolbox (Tadel et al., 2011). A band-pass filter between 1 and 45 Hz was applied to the EEG data to remove power line contamination and low frequency artifacts. EEG data were manually examined by a careful visual inspection to detect and remove artifacts confirmed by an EEG expert (SP), taking into account the following steps: (1) Identifying channels that are contaminated by noise or artifacts (flat channels, impedances checks, jumps, ocular, muscle activity or excessive movement, etc). (2) Interpolating channels marked as bad using spherical splines (Perrin et al., 1989). A maximum of 1 channel was interpolated from a trial and if more channels were found as bad the whole trial was rejected from the analysis. (3) Identifying a trial as good if the average amplitude of the channels was less than 200 μV (Cohen, 2014; Komosar et al., 2022). Also, we considered trials that showed only continuous and synchronous EEG patterns since all the infants were full term around 39 weeks (Eisermann et al., 2013; St Louis et al., 2016). Higher frequencies, from beta to gamma range, were not included in the analysis to avoid contamination with muscle activity. The remaining artifact-free data were segmented into four-second epochs, according to the audio/distress segmentation criteria to the following conditions: resting, cry, and distress. EEG data analysis was performed for the following classical frequency bands: delta (ẟ: 1-4 Hz), theta (θ: 4-8 Hz) and alpha (α: 8-12 Hz). Additionally, the power spectrum of each EEG sensor was computed by using Welch’s periodogram method (Welch, 1967). For each sensor, relative power was calculated by normalizing the power at each frequency by total power over the 1–45 Hz range.

To quantify the relative power changes across conditions with respect to the resting state, the total relative power of the frequency bands analyzed was considered as 100%, and the percentage of relative power for each frequency band was calculated for each sensor and all the conditions.

### 2.5. Near-infrared spectroscopy pipeline

#### 2.5.1. Data acquisition

Root O3™ (Masimo, United States) was the equipment selected for NIRS data acquisition. This device uses NIRS forehead sensors to enable measuring regional hemoglobin oxygen saturation (rSO_2_), i.e., the central oxygenation level. Functional arterial hemoglobin oxygen saturation (SpO_2_), i.e., the peripheral oxygenation level and pulse rate (PR-bpm), i.e., the heart rate signal are continuously and non-invasively monitored with a fingertip sensor on the newborn.

#### 2.5.2. Data processing

rSO_2_, SpO_2_, and PR-bpm data were collected every 2 s and saved by the device. Later, these variables were exported offline and analyzed in Python 3. NIRS data that were characterized by a standard deviation lower than 0.5 were not considered in the analysis to eliminate errors from the data acquisition process. Also, the interquartile range (1.5*IQR) method was used to remove outliers. The remaining non-rejected data were segmented into normal cry, distress and resting time episodes based on the timestamps obtained in the audio segmentation criteria. The 15 s preceding and following each segment were discarded. In addition, a low band-pass filter was applied to the corresponding CE intervals removing SpO_2_ values whose mean were lower than 80% (Lu et al., 2014), rSO_2_ lower than 50% (Lian et al., 2020), or PR-bpm lower than 70 beats per minute (Kliegman and Geme, 2019) to eliminate noise and errors derived from newborn’s movements.

### 2.6. Facial expression and body movement analysis

Nowadays, neonatologists use common tools to measure distress levels in newborns from a qualitative perspective, especially assessing crying, facial expressions, and body movements. Among them, the COMFORT scale allows for assessing distress levels, states, sedation, and pain in nonverbal pediatric patients, being cry characteristics part of the assessment (Van Dijk et al., 2000; Wielenga et al., 2004). The COMFORT scale was adapted to Spanish, and it has been shown to be a valid and reliable tool (Cronbach alpha coefficient of 0.785 for newborns) to assess comfort levels in a group of children admitted to a Spanish Intensive Care Unit (Bosch-Alcaraz et al., 2020, 2022). The COMFORT scale has been used to qualitatively evaluate the video recordings of facial expressions and body movements during each session and to identify the levels of cry distress.

#### 2.6.1. Data acquisition and processing

A high-quality video recording of the newborn was acquired for each session ensuring the registration of facial expressions and body movements following a standardized protocol. Afterwards, two experts reviewed (AL, IAP) and assessed the newborns individually according to the COMFORT scale for each CE on the video. In case of disagreement between the experts, a third reviewer (AP) was asked to present their evaluation. The aspects evaluated include six sections: alertness, agitation, crying, body movements, muscular tone, and facial tension. Each section can be rated from 1 (calm infant) to 5 (stressed infant) and the total distress score of each CE ranges from 6 to 30, with larger score values indicating a higher arousal threshold.

### 2.7. Statistical analysis

Statistical analysis was performed using Matlab r2022a, Graphpad Prism 8 and SPSS22. Comparisons were conducted between resting, crying, and distress conditions for audio, EEG, NIRS, and the COMFORT scale. The Shapiro–Wilk test was applied to verify that data were not normally distributed. Data collection involved spontaneous cry recordings, resulting in imbalanced condition segments. Thus, representative segments were randomly selected for each signal feature (audio, EEG, NIRS).

ANOVA and Tukey–Kramer tests were used to compare audio and NIRS processed data, with bootstrapping (10,000 repetitions) for normality correction. Mann–Whitney U-test were used for EEG and COMFORT scale data pairwise, while Kruskal-Wallis test for comparison for more than 3 conditions. For EEG pairwise comparisons, the Holm-Bonferroni correction method was applied while for the 3 condition comparisons the Dunn’s test was selected.

For an integrative approach, the Spearman (Rho) correlation coefficient was used to correlate all features. Additionally, the Kendall Coefficient of Concordance (W) was calculated to assess the level of agreement between audio features with EEG, NIRS and COMFORT scale. We used Cohen’s interpretation guideline (Cohen, 2013), where W > = 0.5 corresponds to strong agreement effects.

## 3. Results

### 3.1. Deep learning algorithm to identify cry distress levels based on cepstral analysis

**Figure 2:**
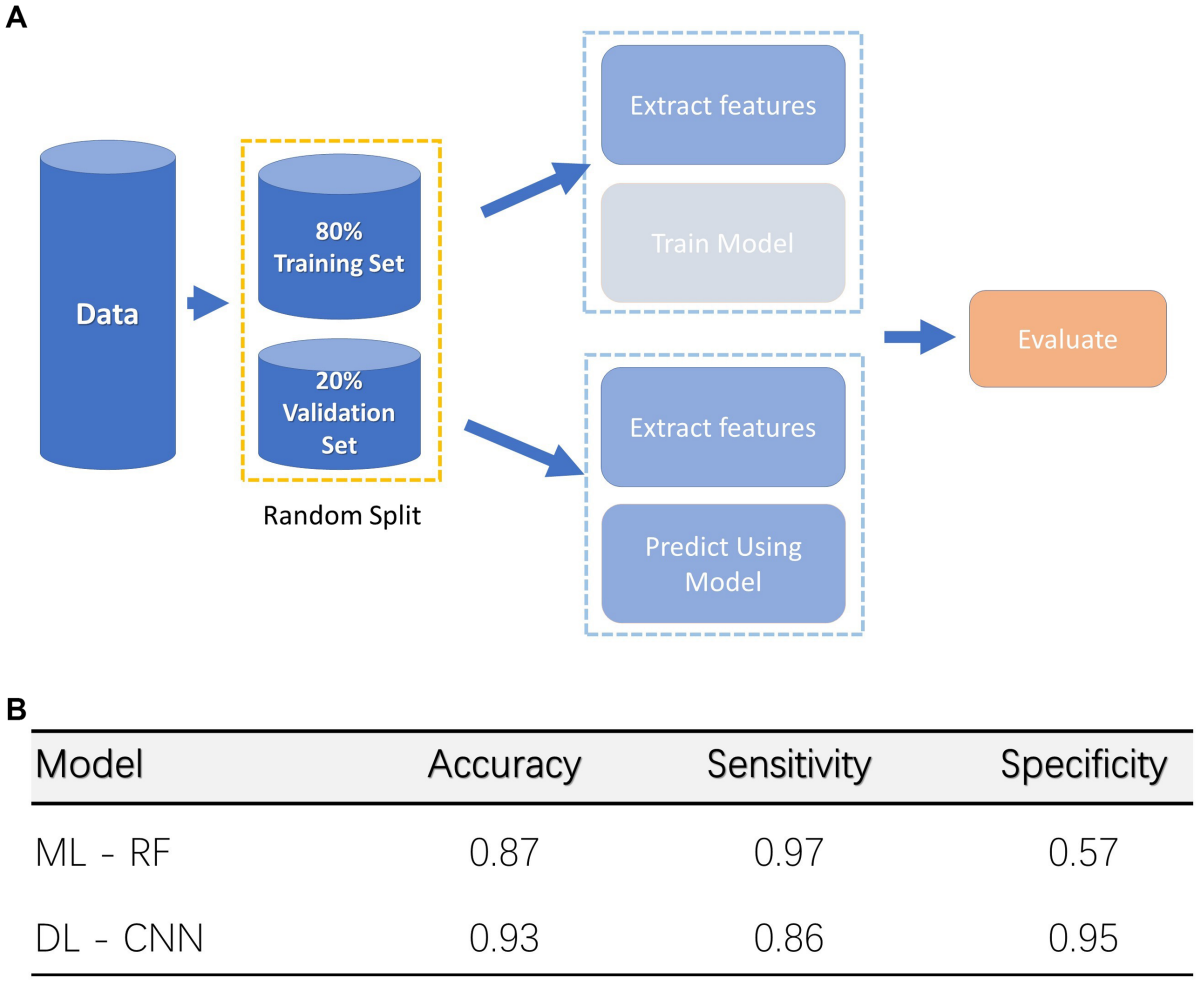
Deep Learning (DL) and Machine Learning (ML) Models. **(A)** Classification procedure for both Machine and Deep Learning models. **(B)** Accuracy for both models, specificity, and sensitivity are also indicated.

The comparison of ML and DL techniques to automatically and objectively evaluate the manual segmentation of the cry recordings and therefore identify different cry levels (Figure 2A) is presented in the current section.

Through the manual segmentation we were able to identify 1,473 cry CU, and 491 distress CU. This dataset was divided into training (1,572 CU) and validation (392 CU) sets to train a classifier. A random split approach has been applied. ML and DL models were trained using the training set. The RF model achieved 89% accuracy, 97% sensitivity, and 57% specificity rates on the validation set discriminating distress vs. non-distress conditions. Instead, the CNN model achieved 93% accuracy, 83% sensitivity, and 95% specificity rates (Figure 2B).

### 3.2. Time and frequency acoustic features characterizing cry distresss levels

The present section shows the results obtained by comparing the cry features extracted with the cepstral analysis and the different cry distress levels identified through the 1964 CU extracted through the manual segmentation.

Table 1 shows the differences between conditions for the acoustic features for time and frequency domain analysis. The time domain analysis showed that the unvoiced CE as its percentage was shorter for distress compared to the cry condition. On the other hand, CryCE exhibited longer periods for cry condition compared to the presence of distress.

**Table 1:** Audio features characteristics (Time and Frequency Domain Analysis) and statistically significant differences among conditions (Cry and Distress conditions).

Feature	Cry	Distress	Statistics	Value of *p*
Acoustic				
Number of CEs	40	21	–	–
Number of CUs	1,473	491	–	–
Time Domain				
Unvoiced CE	19.446 ± 17.510	14.825 ± 9.193	1,276	0.589
Unvoiced CE (%)	0.395 ± 0.163	0.353 ± 0.177	1,320	0.227
Cry CE	30.833 ± 31.882	30.152 ± 16.749	1,190	0.452
Cry CE (%)	0.605 ± 0.163	0.647 ± 0.177	1,160	0.227
Frequency Domain				
F0(mean)	477.563 ± 109.396	412.587 ± 109.124	11.419	0.001*
F0(min)	292.807 ± 112.566	221.517 ± 71.113	13.183	0.001*
F0(max)	717.339 ± 250.428	752.683 ± 283.166	−2.619	0.015*
F0(std)	94.230 ± 64.355	141.050 ± 86.254	−12.749	0.001*
F1(mean)	1428.963 ± 406.158	1630.672 ± 470.433	−9.148	0.001*
F2(mean)	3557.709 ± 448.793	3739.816 ± 452.544	−7.738	0.001*
F3(mean)	5897.238 ± 487.545	6094.533 ± 458.543	−8.125	0.001*
High-pitch(F0 > 800 Hz)	0.029 ± 0.122	0.047 ± 0.125	−2.886	0.005*
Hyper-phonation(F0 > 1,000 Hz)	0.012 ± 0.079	0.023 ± 0.081	−2.590	0.011*
HNR	11.880 ± 4.381	6.662 ± 3.300	23.862	0.001*
Jitter	0.016 ± 0.011	0.022 ± 0.013	−10.437	0.001*
Shimmer	0.113 ± 0.044	0.143 ± 0.041	−13.228	0.001*

ANOVA and Tukey–Kramer tests were used for post hoc comparisons and a bootstrapping procedure was repeated 10,000 times to correct for normality and unbalanced categories. F values and *p*-values are shown in the two last columns. Data are presented as mean ± std. * is referred as statistically significant (*p* < 0.05) and ** as statistically highly significant (*p* < 0.001).

Moreover, F0 (mean), F0 (min) and HNR decreased in the distress condition compared to the cry one. An increase in features such as F0 (max), F0 (std), F1, F2, F3, high-pitch (F0 > 800 Hz) and hyper-phonation percentage (F0 > 1,000 Hz), Jitter, and Shimmer were found for distress compared to cry condition (see Table 1).

### 3.3. Patterns in neurophysiological data for different cry distress levels

**Figure 3:**
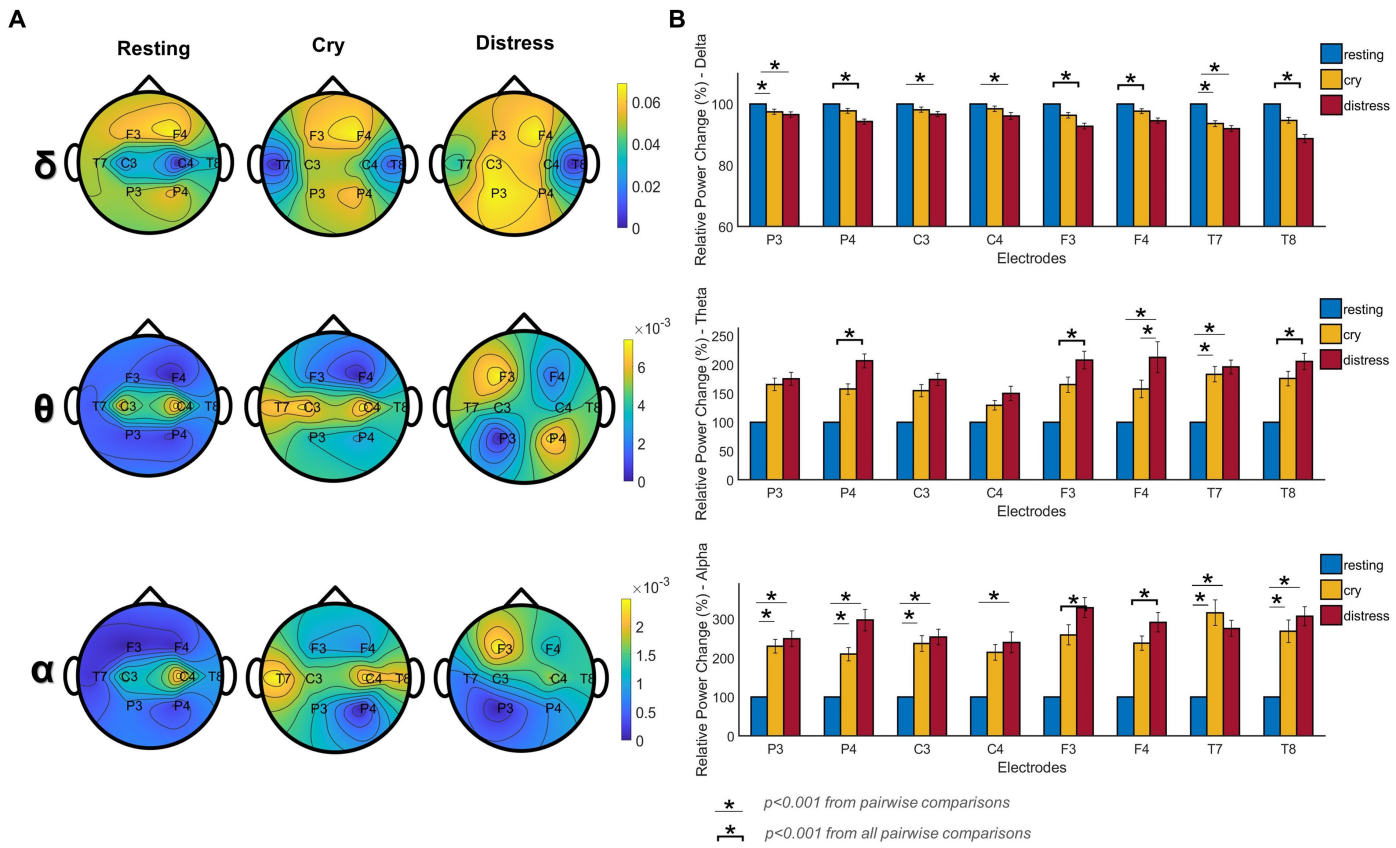
Differences in power spectrum for resting, cry, and distress conditions (*n* = 295 segments, for both conditions, n was balanced using random sampling), were obtained by applying a Kruskal-Wallis test with Dunn’s test (for post-hoc comparisons). **(A)** Topographic EEG maps of relative power distribution for delta (ẟ), theta (θ), and alpha (α) bands. The upper portion of each map shows the nose (frontal area) and the lower side shows the occipital side. **(B)** Percentage of relative power changes across frequency bands and electrodes for each condition. Specifically, for Figure 3, * and the line below represents a statistically highly significant difference *p* < 0.001 from pairwise comparisons. * and the bracket indicates a statistically highly significant difference *p* < 0.001 for all the pairwise comparisons.

Regarding the EEG findings, the power spectrum analysis showed that the relative power change in the delta band decreased compared to the resting condition (*p* < 0.001; Figure 3B). For theta and alpha bands, an increase of the relative power change compared to the resting condition was observed. Additionally, Figure 3A shows the topological distribution of the relative power for all conditions for delta, theta, and alpha bands. For different cry distress levels, the resting condition attenuated, and the distribution of the power varied. The cry condition showed in the delta band a frontal relative power distribution. The distress condition showed a fronto-parietal pattern compared to the resting condition in delta and theta bands, and a frontal relative power distribution for alpha band.

Figure 3B depicts the percentage of change in relative power for the different cry distress levels studied. In the delta band, all electrodes presented statistical differences (*p* < 0.001) showing a decrease in the percentage of change in the relative power and the mean percentage of change for cry was −3.15% and − 6.27% for distress conditions compared to resting (100%). An increase in the percentage of change can be observed in the theta band (*p* < 0.001). The mean percentage of change for the cry condition was 66.54 and 93.67% for distress compared to resting. All electrodes on alpha showed statistically significant differences in the percentage of change (*p* < 0.001). The mean percentage of change for cry was 166.55 and 215.69% for distress compared to resting.

**Figure 4:**
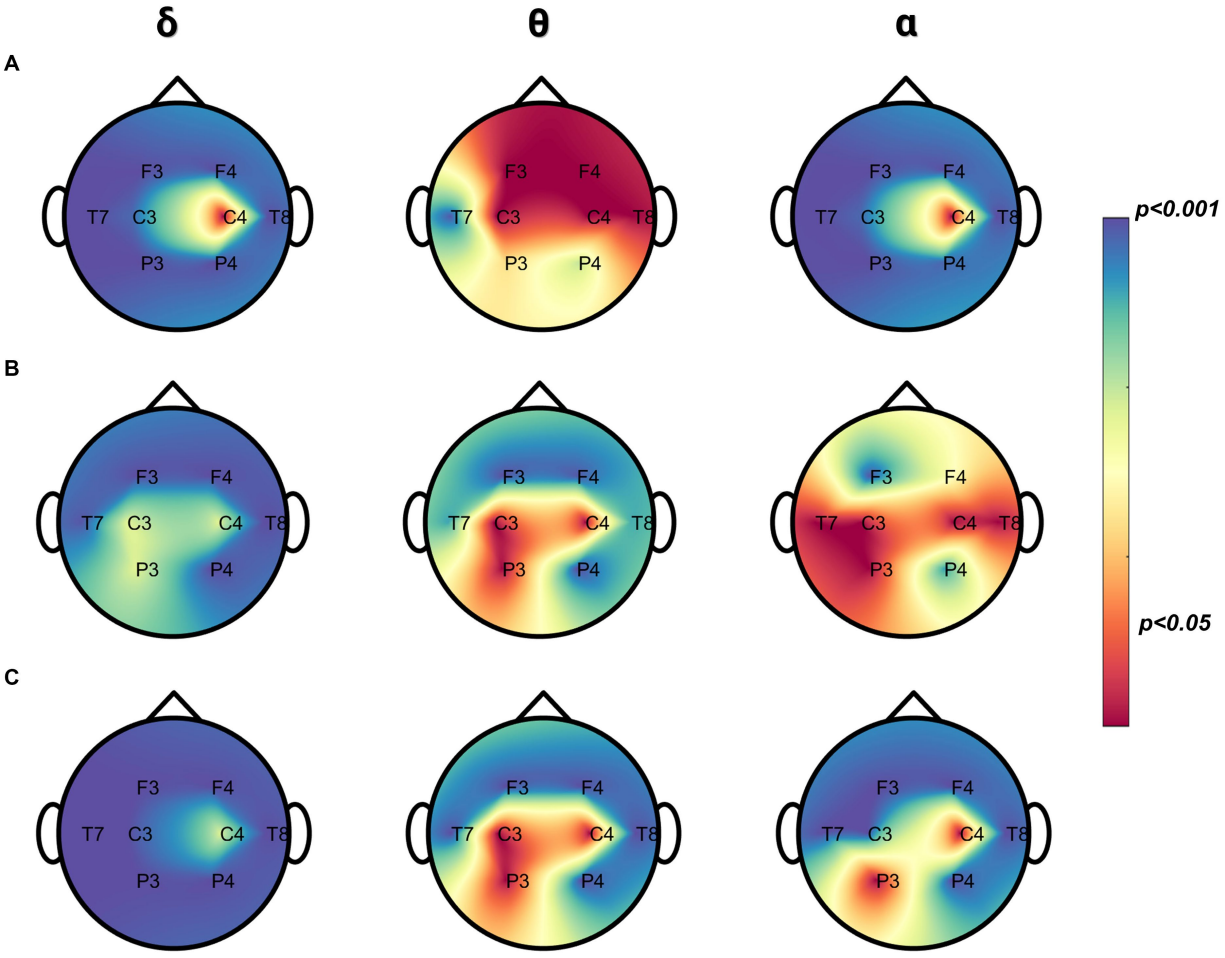
Pairwise comparisons between cry, distress, and resting in relative power. **(A)** Differences between cry and resting (*n* = 295 segments, for both conditions, n was balanced using random sampling) were obtained by the Mann–Whitney test. **(B)** Differences between distress and resting (*n* = 180 segments, for both conditions–n was balanced using random sampling) were obtained by the Mann–Whitney test. **(C)** Differences between cry and distress (*n* = 180 segments, for both conditions, n was balanced using random sampling) were obtained by the Mann–Whitney test. The color bar is displayed as a family-wise corrected significance level of 1–value of *p*: the blue darker color depicts a higher statistically significant difference between pairwise comparisons and the red color the opposite.

Furthermore, a significant and diffuse pattern can be observed in the whole head (Figure 4, a-b-ẟ-α) for delta and alpha band when comparing the resting and cry conditions. Antero-posterior statistically significant differences were found comparing different cry distress levels in the delta and theta bands while the alpha band showed mostly frontal differences (Figure 4, b-θ-α, c-ẟ-θ-α). In theta band, a posterior pattern of differences occurred comparing resting and cry conditions (Figure 4, a-θ). Supplementary Table 1S (see Supplementary material) reports the results of the statistical analysis.

Briefly, the distress condition, acoustically associated with high spectral content and intensity over time, presented higher percentage changes in relative power in the theta and alpha bands, and conversely lower in the delta band compared to the cry and resting conditions.

### 3.4. Variation in the oxygenated hemoglobin level during the newborn arousal state

**Figure 5:**
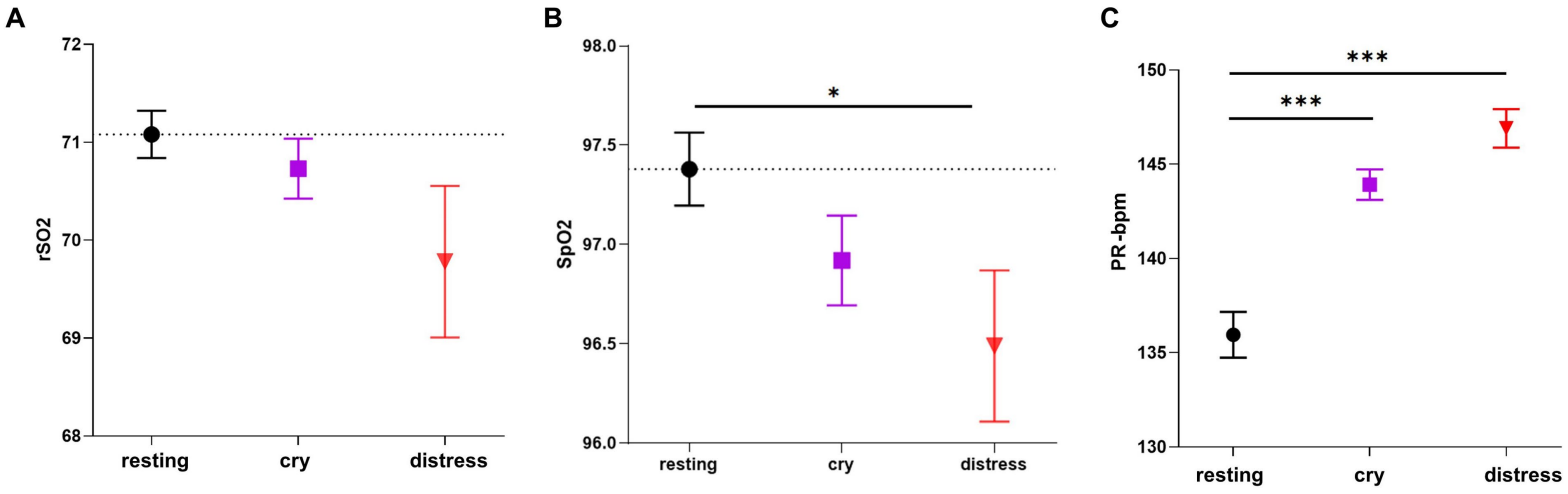
Comparisons in rSO2, SpO2, and PR-bpm among the three conditions. **(A)** rSO2 differences among resting (*n* = 441 segments), cry (*n* = 272 segments), and distress conditions (*n* = 140 segments). **(B)** SpO_2_ differences among resting (*n* = 361 segments), cry (*n* = 295 segments), and distress conditions (*n* = 150 segments). **(C)** PR-bpm differences among resting (*n* = 421 segments), cry (*n* = 295 segments), and distress conditions (*n* = 153 segments). ANOVA and Tukey–Kramer tests were applied for *post hoc* comparisons and the bootstrapping procedure repeated 10,000 times was applied to correct for normality and unbalanced categories. Resting is displayed as a black circle, cry as a purple square, and distress condition as a red triangle. The dotted line for each variable represents the mean value for the resting condition. *** indicates *p* < 0.001 and * indicates *p* < 0.05. Data are presented as mean ± standard error mean.

Figure 5 shows the differences between the regional and functional arterial hemoglobin levels together with the pulse rate in the different newborn conditions. rSO_2_ decreased in the cry and distress condition compared to the resting condition (Figure 5A) even though no statistical differences were found. SpO_2_ also decreased in the cry and distress conditions (*p* < 0.05) compared to the resting condition (Figure 5B). PR-bpm increased during cry (p < 0.001) and distress (*p* < 0.001) conditions compared to resting (Figure 5C). From a descriptive perspective, when high spectral content and intensity are present acoustically, we noticed a trend of SpO_2_ and rSO_2_ decreases accompanied with a statistically significant increase of the PR-bpm. Supplementary Table 1S (see Supplementary material) shows significant differences between rSO_2_, SpO_2_, and PR-bpm.

### 3.5. Behavioral changes determined by the distress in cry acoustic features

**Figure 6:**
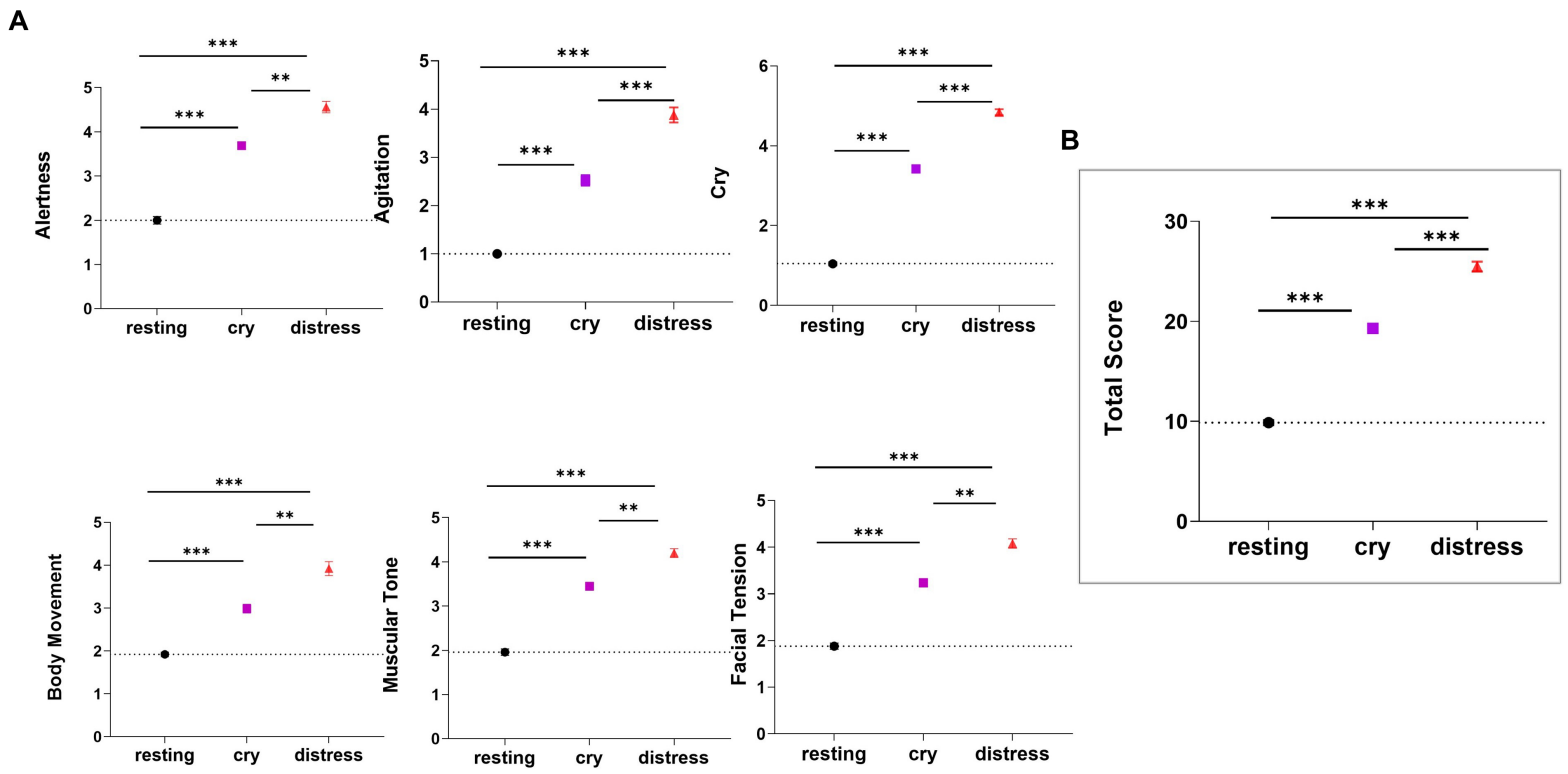
Comparisons of the COMFORT scale scores among conditions (resting: *n* = 24 segments, cry: *n* = 67 segments, and distress: *n* = 25). **(A)** Alertness, Agitation, Cry, Body Movement, Muscular Tone, Facial Tension scores and **(B)** Total scores are reported. The Kruskal-Wallis test along with Dunn’s test (for post-hoc comparisons) were used. The dotted line for each variable represents the mean value for the resting condition. *** indicates *p* < 0.001 and * indicates *p* < 0.05. Data are presented as mean ± standard error mean.

Figure 6 shows the differences between all items within the COMFORT scale for different cry distress levels. Higher scores were found in the distress condition for all the features analyzed compared to cry and resting conditions. Supplementary Table 2S (see Supplementary material) shows detailed values for the statistical significance comparison among conditions.

### 3.6. Integrative approach between audio features and neurophysiological signals

With the aim to explore to what extent the audio features of the different cry distress levels were associated with the neurophysiological and behavioral variables analyzed in this study, we applied a Spearman correlation analysis and Kendall’s coefficient (W) of concordance.

**Figure 7:**
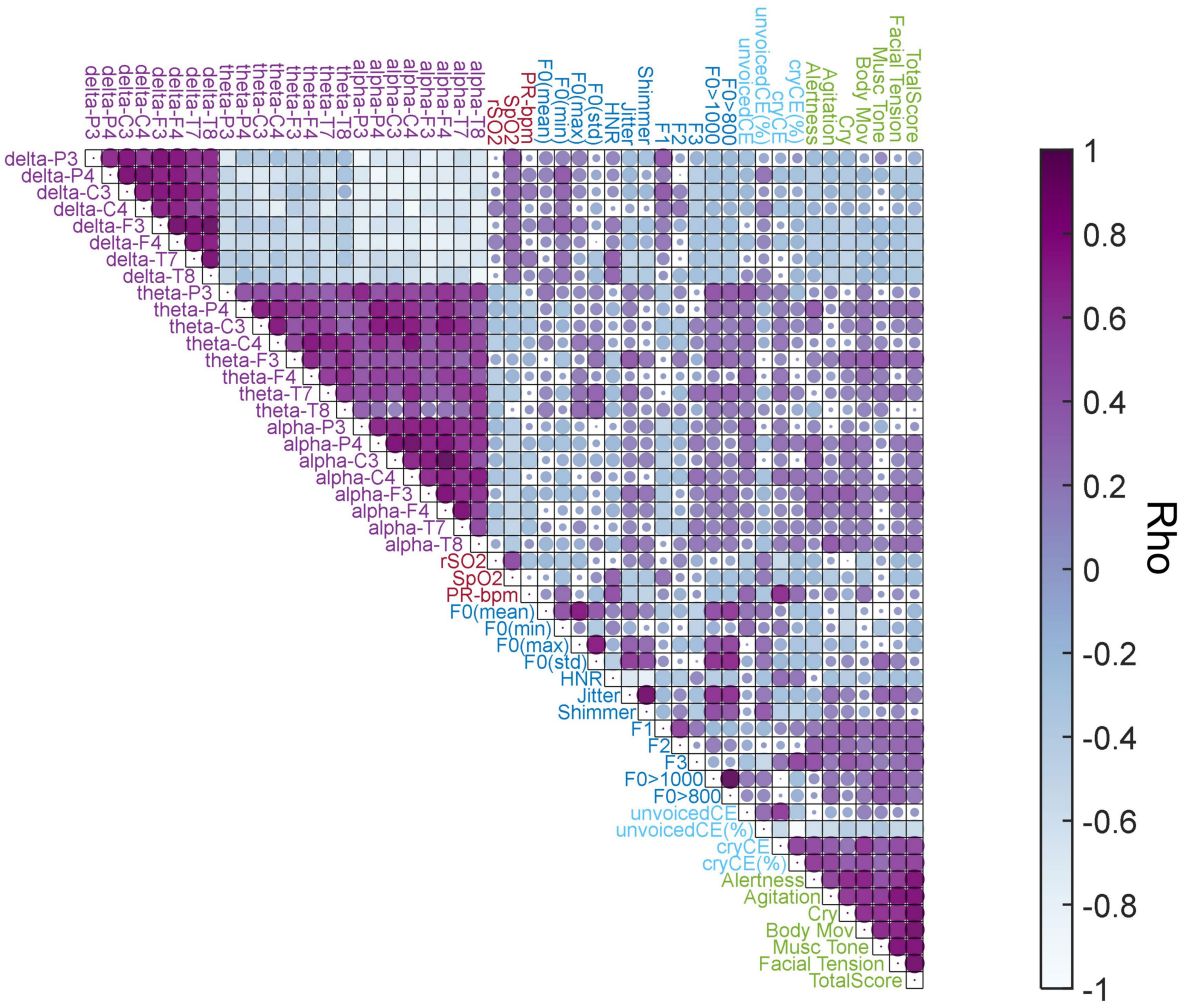
Correlation Matrix. Spearman Correlation coefficients (rho) among acoustic features, EEG relative power frequency bands, NIRS, and COMFORT scale. The colormap represents the rho values. The darker purple color indicates positive correlations and the blue light color the negative ones. Circle size indicates the statistical significance level (1-value of *p*), thus, a bigger circle size represents higher statistically significant levels and a smaller size indicates the opposite. Feature group labels: light blue is used for cry temporal features; darker blue for cry frequency features; light purple for EEG relative power frequency bands; light red for NIRS features; and light green for the COMFORT scale scores.

Figure 7 shows the correlation matrix between all features analyzed. Audio features such as F0 (min) (ẟP4: *R* = 0.42–*p* = 0.04) and F1 (ẟP3: *R* = 0.43–*p* = 0.03, ẟC3: *R* = 0.42–*p* = 0.03) correlated positively to the EEG relative power in delta band, respectively. However, we found negative correlations when Jitter (ẟT7: *R* = -0.49–*p* = 0.01), Shimmer (ẟT7: *R* = -0.45–*p* = 0.02) and F3 (ẟP4: *R* = -0.42–*p* = 0.03, ẟC3: *R* = -0.40–*p* = 0.04) are compared to the delta band power. On the other hand, Jitter electrode F3: (*R* = 0.42–*p* = 0.03), F0 > 800 Hz (θP3: *R* = 0.41–*p* = 0.04) and F0 > 1,000 Hz (θP3: *R* = 0.45–*p* = 0.02) positively correlate with EEG on theta band power, respectively. Contrary to delta band, F1 (ẟP3: *R* = –0.49–*p* = 0.01, ẟC3: *R* = –0.40–*p* = 0.04) correlated negatively with theta band power.

On NIRS, we found a negative correlation between the rSO_2_ with cryCE (*R* = –0.54–*p* = 0.005) and a positive one between PR-bpm and cryCE (*R* = 0.67–*p* = 0.0003). Additionally, delta band power correlated positively with SpO_2_ (ẟP3: *R* = 0.43–*p* = 0.03). Furthermore, we found a negative correlation in theta band power and rSO_2_ (θC4: *R* = –0.41–*p* = 0.04) and between alpha band power and SpO_2_ (ɑP3: *R* = –0.41–*p* = 0.04, ɑP4: *R* = –0.45–*p* = 0.02, ɑF3: *R* = –0.49–*p* = 0.01, ɑF4: *R* = –0.46–*p* = 0.02 and ɑT7: *R* = -0.48–*p* = 0.01).

For the COMFORT scale, the percentage of cryCE correlated positively with all the scores from the COMFORT scale (*p* < 0.01). On the other hand, we found negative correlations between the percentage of unvoicedCE and most of the COMFORT scale scores (*p* < 0.01). For a detailed description of all statistically significant correlations found related to these comparisons and other interesting but non statistically significant correlations see Supplementary Tables 3S, 4S.

**Figure 8:**
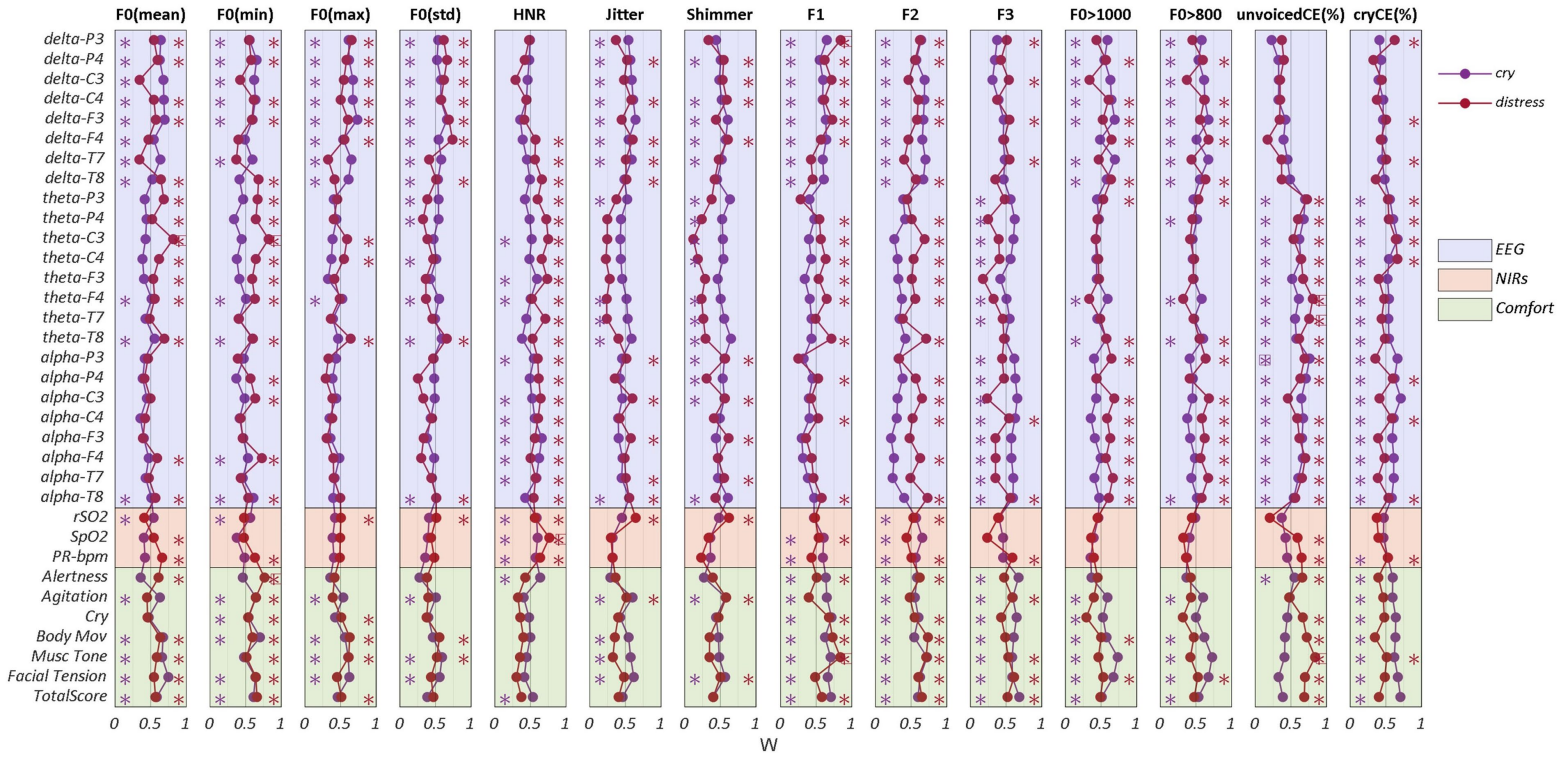
Concordance Analysis. Kendall coefficients (W) between acoustic features and EEG, NIRS, and COMFORT scale for cry (purple) and distress (red) conditions. * indicates W coefficients greater than 0.5. W coefficients greater than 0.7 are framed with a rectangle. To group the variables within each feature (EEG, NIRS, and COMFORT scale), different colors were set in the figure (light purple for EEG, light red for NIRS, and light green for the COMFORT scale).

To measure the level of agreement among audio features, EEG and NIRS features, and the COMFORT scale scores during cry and distress conditions, the concordance coefficient W was computed. Figure 8 shows W coefficients for the cry (purple) and distress (red) conditions, an asterisk identifies the W values greater than 0.5 indicating strong agreement levels among features.

Most of the EEG features exhibited strong levels of agreement with the audio features such as F0 (mean, min, max, std), Jitter, Shimmer, F1, F2, F0 > 800 and F0 > 1,000 with delta band power for cry and distress conditions. HNR, cryCE (%) and unvoicedCE (%) showed higher levels of agreement with theta and alpha band power in both cry and distress conditions. Additionally, F3, the percentage of high-pitch (F0 > 800) and the percentage of hyper-phonation (F0 > 1,000) presented stronger levels of concordance with the alpha band power.

F0 (mean and min), HNR, F1, F2 and cryCE (%) exhibited a strong level of concordance with theta band power, especially for distress. The higher values of agreement (W > 0,75) were found for F0 (mean and min) with theta band power (electrode C3), unvoicedCE (%) with theta band power (electrodes F4 and T7) in the distress condition and alpha band power (electrode P3) in cry condition.

Regarding NIRS features, HNR exhibited the strongest level of concordance in both cry and distress conditions for rSO_2_, SpO_2_ and PR-bpm.

Concerning the COMFORT scale scores, the stronger agreements are present on F0 (min) for the distress condition and the resonance frequencies (F1, F2, andF3), hyper-phonation and cryCE (%) in the cry condition.

## 4. Discussion

This study presents an innovative multimodal analysis during different cry distress newborn conditions. Our findings showed, for the first time, that cry acoustic features are correlated with EEG, NIRS, facial expression and body movement changes, supporting cry research studies that want to prove the potential use of cry analysis as a clinical biomarker to describe the infant’s health status.

Additionally, we demonstrated that there are statistically significant differences among the features related to the three newborn conditions (i.e., cry, distress, and resting). Finally, we have also developed a DL algorithm as an objective and automatic approach to identify distress cries supporting clinicians on the assessment of the infant’s well-being.

Limited research has been conducted to understand infant cry as a reflection of complex neurophysiological and behavioral functions. Previous studies investigated correlations between newborn cry acoustic features such as F0 and NIRS (Orlandi et al., 2012), neonatal facial expressions (De Melo et al., 2014), EEG (Futagi et al., 1998), or body movements (Orlandi et al., 2015) separately. However, no studies have been conducted to concurrently analyze cry and neurophysiological and behavioral signals to different newborns’ cry distress levels.

Our results suggested that higher cry distress levels in newborns represented more F0 changes, high-pitched and hyper-phonated cries along with tendencies of higher Jitter and Shimmer and lower HNR, higher amount of cryCE and less unvoiced periods, decrease delta activity and increase theta and alpha activation, higher heart rate, lower cerebral and body oxygenation, and higher scores on the COMFORT scale assessment of the body/face expressions. These results matched with the scant studies (Porter et al., 1988; Shinya et al., 2016) investigating the relation between vagal function and the F0 of infant crying, even in typically developing infants. This is in line with Zeskind’s findings (Zeskind et al., 1985) where cries with a faster repetition rate, shorter cry expirations or pauses, and higher F0 values may elicit more urgent caregiver responses than other vocalizations with less intense acoustic characteristics. Also, our results matched the limited literature on Jitter, Shimmer, HNR or excessive crying when studying irritable infants (Fuller et al., 1994) or dysphonation in adults (Teixeira and Fernandes, 2015). In a summary, our findings were consistent with the assumption that the myelinated branch of the vagus system is involved in both the regulation of heart rate and laryngeal muscles, suggesting that vagal influence on the heart may reflect vagal output to the laryngeal muscles, related to the F0 of infant crying (Shinya et al., 2016). In fact, the audio features extracted from the time domain analysis such as cryCE correlated negatively with rSO_2_ and positively with PR-bpm. Moreover, several items from the behavioral COMFORT scale were associated with F0 (mean), F1, F3, hyper-phonation (F0 > 1,000 Hz), unvoicedCE and cryCE percentages. These results were also coherent with the findings from Craig et al. (2001) enhancing the association of the state of arousal of the infant cry acoustics with physiological measures such as higher cardiac vagal tone and lower oxygen levels combined with behavioral signs of cry distress such as facial tension, rigidity, or vigorous body movements.

Regarding neurophysiological signals, two previous (Futagi et al., 1998; Maitre et al., 2017) studies analyzed cry episodes and EEG brain activity as mentioned in the Introduction section. However, these studies do not delve into the dynamics of the cry or the different cry distress levels over different frequency bands, or do they add extra variables that allow the identification of other patterns.

In our study, we proved that the delta band relative power of the different distress levels decreased compared to the resting state condition. Delta band in a predominant frequency with diffuse activity over central and occipital regions during wakefulness of a newborn (Eisermann et al., 2013). Therefore, it is logical that while other types of electrical activity decrease, resting activity increases in this frequency band.

Moreover, theta and alpha bands depicted an increase in the percentage relative power change compared to the resting condition (more than 60% for theta band and more than 100% for alpha one) over frontal–parietal and temporal areas. These increases in power over different cry distress levels suggest the association between these bands and stress episodes (Norman et al., 2008; Seo and Lee, 2010).

Furthermore, for frequency audio features, F0 (min), high pitch (F0 > 800 Hz), hyper-phonation (F0 > 1,000 Hz), jitter and shimmer correlated with delta and theta bands power in EEG, mainly in frontal, temporal and parietal electrodes. Other features such as F0 (mean), F0 (std), HNR, cryCE (%) and unvoicedCE (%) show evident trends in the same frequency bands. Moreover, some electrodes in delta, theta, and alpha bands correlate with the values of the COMFORT scale. According to the literature related to cortical activation in adults (Welch, 1967) and newborns (Eisermann et al., 2013), the correlations that we found enhance the fact that more intense cry vocalizations characterized by higher spectral values represent an increase of brain activity in theta and alpha band and a decrease in delta band power, implying more agitation for the newborn.

Additionally, it is important to highlight that, to the best of our knowledge, to this date there are no studies that have used DL with a CNN approach for the classification of the different cry distress levels of the newborn achieving robust and high accuracy results. Most of the literature assessing infant cry distress levels is based on ML classification techniques (Xie et al., 1996; Parga et al., 2020) with less than 90% of accuracy rates. Our DL approach obtained 93% accuracy, 83% sensitivity, and 95% specificity, showing better performance in identifying distress and non-distress infant cries and supporting the validation of our audio manual segmentation. These results highlight the potential of AI tools for screening or decision support in the healthcare system automatically and objectively supporting clinicians on the assessment of stress or pain in the neonatal unit (e.g., after surgical interventions) or primary care settings (e.g., in pediatric routinary visits or follow up clinics).

Nevertheless, this exploratory study presents some limitations. The main ones are related to the small sample size presented and the low density of EEG (i.e., only 8 electrodes were recorded) and NIRS (only one frontal electrode was used) systems. Despite this limitation, we were able to identify clear patterns of brain activity, statistical differences and associations were found among features and newborns’ conditions. Given the restricted sample size, additional research is required to substantiate the significance of solely utilizing cry acoustic features within a predictive model for monitoring the health status of newborns. Another limitation of our study is linked to the difficulty experienced during data acquisition because infant recordings are usually affected by noise artifacts, either muscular due to neonatal movement or contamination due to environmental noise. In addition, the analysis of the NIRS and EEG while crying can be quite challenging due increase in excessive movement and muscle activity from the infant. In our specific scenario, the restriction of infant movement becomes notably intricate, as our intent is to assess all variables within a naturalistic environment. Consequently, this inherent limitation prompts a deliberate selection of methodological strategies designed to enhance the signal’s quality. Lastly, we were not able to collect balanced data samples for each condition due to the nature of spontaneous crying. In fact, infants usually cry less often in painful or stressful situations. As such our data samples are limited.

Future studies will focus on expanding the sample size and utilizing denser EEG systems to explore the neurophysiological sources associated with different cry distress levels and their correlation with prematurity and pathological indicators. Specifically, we aim to increase the number of healthy term infants and include preterm and pathological infants in a longitudinal multicentric study. This approach will allow us to replicate and extend the analysis presented in this manuscript, comparing data from diverse sub-cohorts to validate the objective nature of infant cry as an indicator of the physical, emotional, and health status of newborns.

## 5. Conclusion

This work characterizes and compares different cry distress levels on acoustic signals with EEG, NIRS and the COMFORT scale scores supporting the idea that different acoustic patterns reflect neurophysiological and behavioral changes related to the newborn arousal state. Furthermore, according to our findings, we have introduced, for the first time, an automated classifier based on a Deep Learning algorithm capable of detecting varying levels of cry distress. This classifier emerges as a potent tool that could greatly facilitate the objective assessment of an infant’s well-being status.

In conclusion, the present study identifies and provides important evidence to cover an existing literature gap related to the multimodal association of newborn cry acoustics with brain activity, cerebral and body oxygenation, heart rate, facial expression, and body movements. This relationship proves that the acoustical analysis of the infant cry may play a pivotal role to recognize different cry distress levels. Moreover, it strengthens the promising use of infant cry as a biomarker supporting caregivers and clinicians on the early detection of certain pathologies and neurodevelopmental disorders.

## Data availability statement

The datasets presented in this article are not readily available because the data that support the findings of this study are available from Zoundream AG. Data can be available from the authors upon reasonable request, and with the written permission of Zoundream AG. Requests to access the datasets should be directed to ana.laguna@zoundream.com.

## Ethics statement

The studies involving humans were approved by Local Ethical Committee: Hospital Clínic Barcelona (Ref: NeuroCry/HCB/2021/0843). The studies were conducted in accordance with the local legislation and institutional requirements. Written informed consent for participation in this study was provided by the participants’ legal guardians/next of kin.

## Author contributions

AL: Conceptualization, Data curation, Formal analysis, Funding acquisition, Investigation, Methodology, Project administration, Resources, Software, Supervision, Validation, Writing – original draft, Writing – review & editing. SP: Conceptualization, Data curation, Formal analysis, Investigation, Methodology, Supervision, Validation, Visualization, Writing – original draft, Writing – review & editing. IA-P: Data curation, Formal analysis, Investigation, Methodology, Software, Writing – original draft. JZ-V: Methodology, Supervision, Writing – review & editing. AP: Conceptualization, Investigation, Resources, Supervision, Writing – review & editing. ÀB: Formal analysis, Methodology, Software, Writing – review & editing. PP: Conceptualization, Methodology, Writing – original draft. CP: Data curation, Resources, Writing – review & editing. OG-A: Conceptualization, Investigation, Project administration, Resources, Supervision, Writing – review & editing. SO: Conceptualization, Investigation, Methodology, Supervision, Writing – review & editing.

## Funding

The authors declare financial support was received for the research, authorship, and/or publication of this article. This research has been funded by Zoundream AG together with the Swiss State Secretariat for Education, Research and Innovation (SERI), the Swiss Innovation Agency (Innosuisse) and NEOTEC (SNEO-20211305).

## Conflict of interest

AL, SP, ÀB, and PP were employed by Zoundream AG. AL is also co-founder of the company and owns stock in Zoundream AG. SO and JZ-V receive compensation for the collaboration as members of the scientific advisory board of Zoundream AG. CP salary is funded by Zoundream AG through Fundació Clínic.

The remaining authors declare that the research was conducted in the absence of any commercial or financial relationships that could be construed as a potential conflict of interest.

## Publisher’s note

All claims expressed in this article are solely those of the authors and do not necessarily represent those of their affiliated organizations, or those of the publisher, the editors and the reviewers. Any product that may be evaluated in this article, or claim that may be made by its manufacturer, is not guaranteed or endorsed by the publisher.
