# Supplementary material for: How can cry acoustics associate newborns’ distress levels with neurophysiological and behavioral signals?
Source: Front Neurosci. 2023 Sep 20;17:1266873. doi: 10.3389/fnins.2023.1266873 (PMC10547902; doi:10.3389/fnins.2023.1266873)
Supplement: Supplementary file 1 [file Data_Sheet_1.PDF]

Supplementary Material

This document presents additional details of the analysis performed in the main manuscript.

Methods Supplementary

Participants. Out of this initial sample, 2 newborns were not included, because we did not obtain enough analyzable data due to movement artifacts. The remaining 23 newborns were included in the final analysis. One single session was recorded per subject.

Exclusion criteria. Newborns under medication and/or with congenital malformations, chromosomal abnormalities, hypoxic-ischemic encephalopathy, intraventricular hemorrhage greater than grade 2, and any other type of brain damage, congenital heart disease, siblings with autism spectrum disorders or other neurodevelopmental disorder were excluded from this study.

Data processing

EEG

EEG electrodes. The non-invasive, gel-based electrodes are fixed to the cap and present a very low profile, which makes this cap very comfortable for the newborn (e.g., avoiding excessive rubbing and pressure onto the scalp).

Methods on automatic algorithms for artifacts correction and detection

We did not use an automatic algorithm because most of the methods available (e.g., ICA) are developed for adults' brain signals acquired in normal environments that generate artifacts that are generally easy to detect and correct. In our case and due to the nature of the acquisition of the data, newborns are crying, sometimes crying irritated while recording, so the movements and artifacts generated by that situation are not easy to detect nor correct, so the automatic methods become ineffective.

These standard methods do not work optimally with data from infants or other populations for whom being still for long periods of time is problematic. Also, it is difficult to precisely identify and remove artifacts arising from eye blinks and eye movements in infants because the EEG components resulting from such movements are not as systematic and temporally confined as in adults. This means that identification of eye-related artifacts in infant EEG is problematic by itself. Thus, although it is well known that approaches like ICA are quite effective in removing typical ocular artifacts in adult data, they are often ineffective when applied to infant data. Finally, infants tend to move abruptly and often, which introduces high amplitude artifacts into the EEG signal. Such abrupt movements can cause the temporary loss of good contact between sensors and the scalp in high-impedance systems. These movement artifacts often contaminate only a few electrodes on any one trial, and different movements affect different trials at different times. Accordingly, they cannot be modeled as sources across trials and, therefore, they cannot be removed using ICA.

Thus, in our study we use a careful and manual visual inspection of the data discarding those trials containing artifacts and keeping the clean ones to ensure the best data quality for the posterior analysis.

__Results Supplementary__

Statistical differences for the different measures analyzed.

Table 1S. EEG relative power and NIRS statistical differences for resting, cry and distress conditions.

Feature	Resting			Cry			Distress			F			p-value		
**EEG**	**δ**	**θ**	**α**	**δ**	**θ**	**α**	**δ**	**θ**	**α**	**δ**	**θ**	**α**	**δ**	**θ**	**α**
P3	0.071 ± 0.007	0.006 ± 0.003	0.001 ± 0.001	0.066 ± 0.011	0.007 ± 0.004	0.002 ± 0.002	0.064 ± 0.011	0.007 ± 0.004	0.002 ± 0.002	14.0057	4.9551	18.7282	<0.0001	0.083	<0.0001
P4	0.071 ± 0.006	0.006 ± 0.003	0.001 ± 0.001	0.067 ± 0.009	0.007 ± 0.004	0.002 ± 0.001	0.064 ± 0.009	0.008 ± 0.004	0.002 ± 0.001	43.2044	32.760	34.4334	<0.0001	<0.0001	<0.0001
C4	0.069 ± 0.006	0.007 ± 0.003	0.002 ± 0.001	0.066 ± 0.01	0.007 ± 0.004	0.002 ± 0.002	0.064 ± 0.01	0.008 ± 0.004	0.002 ± 0.002	11.6694	3.1615	24.6718	0.0029	0.2058	<0.0001
C3	0.068 ± 0.007	0.007 ± 0.003	0.002 ± 0.001	0.065 ± 0.011	0.007 ± 0.004	0.002 ± 0.002	0.063 ± 0.011	0.007 ± 0.004	0.003 ± 0.002	8.97477	2.2846	10.6601	0.0112	0.3190	0.0048
F4	0.071 ± 0.006	0.006 ± 0.003	0.001 ± 0.001	0.067 ± 0.01	0.006 ± 0.004	0.002 ± 0.002	0.063 ± 0.01	0.008 ± 0.004	0.003 ± 0.002	54.6460	34.865	77.0336	<0.0001	<0.0001	<0.0001
F3	0.072 ± 0.006	0.006 ± 0.003	0.001 ± 0.001	0.068 ± 0.009	0.006 ± 0.004	0.002 ± 0.002	0.064 ± 0.009	0.007 ± 0.004	0.002 ± 0.002	37.9468	18.353	60.1832	<0.0001	<0.0001	<0.0001
T7	0.071 ± 0.007	0.006 ± 0.003	0.001 ± 0.001	0.061 ± 0.013	0.007 ± 0.004	0.002 ± 0.002	0.061 ± 0.013	0.008 ± 0.004	0.002 ± 0.002	72.2258	39.875	73.6288	<0.0001	<0.0001	<0.0001
T8	0.07 ± 0.007	0.006 ± 0.003	0.002 ± 0.001	0.062 ± 0.012	0.007 ± 0.004	0.002 ± 0.002	0.057 ± 0.012	0.008 ± 0.004	0.002 ± 0.002	66.7946	36.067	59.9334	<0.0001	<0.0001	<0.0001
**NIRS**															
rSO2	71.08±5.09			70.73±5.06			69.78±9.15			2.53			0.08		
SpO2	97.38±3.49			96.92±3.88			96.49±4.66			3.04			0.48		
PR-bpm	135.95±24.97			143.92±13.88			146.90±12.63			23.25			<0.0001		

Table 2S. Differences in the COMFORT Scale for different distress levels.

Feature	Resting	Cry	Distress	F	p-value
**COMFORT Scale**					
Alertness	2.000 ± 0.417	3.687 ± 0.632	4.560 ± 0.650	72.64	<0.0001
Agitation	1.000 ± 0.000	2.522 ± 0.893	3.880 ± 0.781	71.19	<0.0001
Cry	1.042 ± 0.204	3.418 ± 0.741	4.781 ± 0.407	67.53	<0.0001
Body Movement	1.920 ± 0.276	2.985 ± 0.825	3.920 ± 0.814	53.78	<0.0001
Muscular Tone	1.958 ± 0.358	3.448 ± 0.657	4.20 ± 0.5	69.55	<0.0001
Facial Tension	1.880 ± 0.331	3.239 ± 0.676	4.080 ± 0.493	69.85	<0.0001
Comfort Total Score	9.875 ± 1.154	19.30 ± 3.143	25.48 ± 2.434	83.88	<0.0001

Table 3S. Statistically Significant Spearman Correlations (Rho and p-values)

Feature	Correlation With Audio Features	Rho	p-value
EEG	delta-P4 - F0 (min)	0.42087	0.040560
	delta-T7 - Jitter	-0.49652	0.013587
	theta-F3 - Jitter	0.42261	0.039652
	delta-T7 - Shimmer	-0.45130	0.026856
	delta-P3 - F1	0.43391	0.034135
	delta-C3 - F1	0.42522	0.038320
	alpha-P3 - F1	-0.49652	0.013587
	alpha-C3 - F1	-0.40783	0.047901
	delta-P4 - F3	-0.42522	0.038320
	delta-C3 - F3	-0.40957	0.046866
	theta-P3 - F0 > 1000	0.45579	0.025196
	theta-P3 - F0 > 800	0.41729	0.042482
	theta-P3 - unvoiced CE	0.46000	0.023715
NIRS	PR-bpm - HNR	0.48261	0.016914
	PR-bpm - Shimmer	-0.47217	0.019823
	rSO2 - cry CE	-0.54435	0.005959
	PR-bpm - cry CE	0.67304	0.000313
COMFORT Scale	F3 - Alertness	0.49553	0.013805
	unvoicedCE (%) - Alertness	-0.61121	0.001509
	cry CE - Alertness	0.51648	0.009769
	cry CE (%) - Alertness	0.61121	0.001509
	unvoicedCE (%) - Agitation	-0.56669	0.003886
	cry CE - Agitation	0.41712	0.042574
	cry CE (%) - Agitation	0.56669	0.003886
	F0 (mean) - Cry	-0.46509	0.022018
	F0 (min) - Cry	-0.52626	0.008250
	unvoicedCE (%) - Cry	-0.40527	0.049455
	cry CE (%) - Cry	0.40527	0.049455
	unvoicedCE (%) -Body Mov	-0.52183	0.008911
	cry CE - Body Mov	0.63426	0.000873
	cry CE (%) - Body Mov	0.52183	0.008911
	F1 - Muscular Tone	0.42484	0.038511
	F0 > 1000 - Musc Tone	0.43306	0.034530
	unvoicedCE (%) - Facial Tension	-0.51746	0.009606
	cry CE - Facial Tension	0.52158	0.008950
	cry CE (%) - Facial Tension	0.51746	0.009606
	unvoiced CE (%) - Total Score	-0.56294	0.004183
	cry CE - Total Score	0.52667	0.008192
	cry CE (%) - Total Score	0.56294	0.004183

Table 4S. Additional Correlations

Correlation	Rho	p-value
theta-C4 - rSO2'	-0.41130	0.045849
delta-P3 - SpO2'	0.43342	0.034362
alpha-P3 - SpO2'	-0.41601	0.043181
alpha-P4 - SpO2'	-0.45518	0.025418
alpha-F3 - SpO2'	-0.49521	0.013875
alpha-F4 - SpO2'	-0.46910	0.020752
alpha-T7 - SpO2'	-0.48216	0.017032
delta-T8 - Agitation'	-0.42825	0.036819
alpha-T8 - Agitation'	0.42112	0.040426
delta-F3 - Cry'	-0.46689	0.021443
delta-F4 - Cry'	-0.42056	0.040722
delta-T7 - Cry'	-0.47679	0.018491
alpha-F3 - Cry'	0.42371	0.039085
theta-F3 - Musc Tone'	0.50334	0.012166
delta-F4 - Total Score'	-0.40473	0.049790

Regarding other correlations (non-statistically significant - trends)

Although some features were not statistically significant, the correlation matrix (see Figure 7 in the manuscript) depicted some clear trends. In summary, audio features such as HNR and F0 (mean) reflect a positive correlation with the delta band power while F0 (max), F0 (std), and Shimmer with theta band power (mainly on electrodes T7, T8, and F3). On the time domain, cryCE% presents some positive correlations with theta and alpha band power while unvoicedCE% with delta band power. In general, the alpha band presents very similar correlations to the theta band, but it shows less strong correlation on audio features.
